# A Pilot Study of Menstrual Health Education, Attitudes, and Product Access in Rural Honduras

**DOI:** 10.3390/ijerph22030374

**Published:** 2025-03-04

**Authors:** Eleanor Stubley, Janice M. Marshall

**Affiliations:** Department of Biomedical Sciences, College of Medicine & Health, University of Birmingham, Birmingham B15 2TT, UK

**Keywords:** menstrual health, menstrual health education, menstrual products, women’s health, sexual and reproductive health

## Abstract

Research data on menstrual health in Honduras are limited, particularly in rural and ethnic minority areas. This pilot study aimed to assess women’s perceptions of menstrual healthcare in rural Honduran communities, focusing on menstrual health education, access to menstrual products and healthcare, and community attitudes towards menstruation. This study was conducted at a 3-day medical clinic set up by Global Brigades in the rural Potrerillos community. Seventy-three female participants (aged 18–55 years) completed a paper-based survey on menstrual health using a Likert scale. Results are reported as descriptive statistics, including median with interquartile range, and 95% confidence intervals. Main findings were that 73.9% of the participants received menstrual health education predominantly at home, with 25% receiving insufficient education before menarche. Additionally, 52.8% of participants reported a lack of and an inadequate range of menstrual products, while 52.9% experienced menstruation anxiety. These findings suggest that community educational initiatives and increased access to menstrual products could significantly improve the menstrual health of rural Honduran women and help reduce negative menstruation experiences.

## 1. Introduction

The World Health Organisation (WHO) defines ‘health’ as ‘a state of complete, physical, mental and social well-being and not merely the absence of disease or infirmity’ [1]. Menstruation is a fundamental part of a woman’s life and development, but despite this, there has been no agreed-upon definition of “menstrual health” [2]. The Terminology Action Group of the Global Menstrual Collective, acknowledging this problem, defined menstrual health using phrasing similar to that used for general health, but included several indicators relating to the menstrual cycle, such as menstrual health education, provision of accessible information, hygiene and privacy, and access to healthcare and treatment. The report emphasised the impact of menstruation on all areas of life, including cultural, social and political areas [2].

The reported average age for menarche—a woman’s first menstrual period—is 12.4 years, commonly occurring between 10 and 16 years old [3]. Thus, to comply with this new definition, the indicators of attained menstrual health, including access to age-appropriate menstrual health education, to menstrual hygiene products and sanitation services during menstruation, and to necessary medical care and treatment should be widely available. It should also be possible for those experiencing menstruation to be able to live free from discrimination or restrictions from participation in normal activities due to menstruation [2]. However, current research on menstrual health issues is limited, as pointed out by Critchley et al. [4] following a major international convention on menstruation.

Importantly, a systematic review published in 2017 found that restrictions to adequate menstrual health are especially severe in low- and middle-income countries, such that girls are commonly uneducated on menstruation when they reach menarche and practises such as missing school and social activities are common, with girls sometimes staying at home during menstruation [5]. The study reported that girls mentioned that education and advice regarding menstruation were provided predominantly by their mothers, with teachers or healthcare professionals being cited as the least likely source of information. In rural Nepal, 76% of girls reported having no access to menstrual health education. In India and Nigeria, studies have reported that age has an impact on knowledge, with older girls receiving more menstrual knowledge [5]. In a study conducted on 456 girls in Accra, Ghana, 80.2% of girls reported they received knowledge about menstruation from their parents [6]. Similarly, a study in rural Malaysia on dysmenorrhea reported that 62.3% of 1295 girls were educated about menstruation by their mothers [7]. Indeed, in the Global Baseline Report from 2018, only 64% of schools in India were reported to be educating girls on menstrual health [8]. The systematic review additionally reported problems with the content of menstrual health education, with 75% of girls surveyed in China stating their knowledge was inadequate. Menstrual knowledge was also linked with misconceptions, with 82% of girls in rural Nepal reporting a belief that menstruation was a curse. In India and Tanzania, where girls rely on their mothers and female relatives for education, this is commonly after the onset of menarche. A study reported that the quality of education differed depending on parental education level in Nigeria, with girls better prepared for menarche if their parents had tertiary education [5]. The lack of education has been linked to a failure to access menstrual healthcare services for dysmenorrhea and menorrhagia [9].

Further, in a study of 352 adolescent girls in Uganda, 19.7% had missed a minimum of one day of school due to their most recent period: lack of suitable hygiene materials and a significant fear of taunting at school were common reasons for absence [10]. Similarly, in a study conducted in Indonesia, 50% of 1159 adolescent girls responded that their menstrual health management was poor [11]. Girls in urban Kenya indicated a preference for menstrual sanitary pads, with nearly 50% utilising cloths instead due to cost concerns; similar concerns were reported in India [5]. A study in rural Kenya listed the use of cloths, from old clothes to towels, blankets and mattress cuttings, with a minority of the girls using sanitary pads due to costs [12]. A review of developing countries reported that reusable cloths are most commonly used in rural locations, compared to sanitary pads in urban locations [13].

Moreover, group discussions and interviews from six rural schools in Kenya indicated that the most common emotion related to menstruation was shame, with ‘unwanted attention’ sometimes associated with these emotions. Indeed, students in one school reported taunting by male teachers and that they were treated differently once they had reached menarche. Girls reported that the lack of discussion concerning menstruation is linked to a belief that menstruation is ‘bad’, with one student quoting that other school children teased them that they are ‘dirty’. These feelings of shame and secrecy were linked with the girl’s fear, with the study reporting that girls feared starting their period when not at home [12]. The 2017 systematic review reported that a majority of girls felt unprepared mentally for their first menstruation, with an urban-based study in India reporting that 64.7% of girls had a negative reaction to menarche [5].

These studies highlight a range of menstrual health management issues that have all been commonly associated with rural residence and a lack of menstrual health knowledge within a community. A lack of adequate menstrual health can result in negative psychological impacts on women’s mental-health. Indeed, an adolescent study in Mexico reported that increasing menstrual health education resulted in less negative attitudes towards menstruation, while a quasi-experimental study in India reported significant increases in the use of sanitary pads following menstrual health meetings [5]. A 2023 review on period poverty suggested that government and NGOs should implement free menstrual hygiene products in public venues, such as schools, as well as promote the dissemination of menstrual health education through media [9]. In rural communities, community leaders and local organisations will be essential to ensuring that these initiatives are accessible to their communities.

Regarding Honduras, the WHO classified it as a ‘priority country’, in part due to the fractured healthcare system. In 2010, approximately 30.1% of the population had no access to healthcare, with 83% having no medical insurance and the lack of access to healthcare being disproportionately suffered by those living in rural areas and by ethnic minorities [14]. Violence continues to be a problem in Honduras, which is reportedly the most violent country worldwide, with San Pedro Sula and Tegucigalpa ranked first and fifth among the worldwide violent cities [15]. The United Nations (UN) 2019 Human Rights Report reported that approximately 40% of female homicides were due to domestic abuse situations, with the law being ineffective and unenforced [16]. The 1981 Contraceptive Prevalence Survey reported that the birth rate of 6.5 births/women was a decline from that shown in previous data [17], but the UN reported that 25% of girls are pregnant before the age of 19, with the rates of adolescent pregnancy being up to 30% in rural populations [16]. Accordingly, statistics reported in 2001 showed that 25% of Honduran women had sexual intercourse before 16 years of age, with this rate increasing to 66% for women who have had sexual intercourse before 20 years of age [18].

In fact, women’s health rights in Honduras are limited, with all abortions prohibited since 1982, including in circumstances of rape or danger to the mother’s life. In 2021, the Honduran government reinforced these restrictions by incorporating the ban into the constitution. Despite this, approximately 15,000 women received medical care due to abortions in 2017 [19]. Further, publications on menstrual health in Honduras are extremely limited, particularly in rural populations. For example, a qualitative study in Santa Rosa de Copan showed that 56.11% of parents could report the age at which menarche generally begins, which is a positive indicator as girls primarily depend on their mothers for this knowledge [20]. Further, an investigation which assessed the impact of a six-year maternal and child health project in rural Honduras reported improvements in maternal health indicators, including prenatal check-up and institutional births. It was concluded that empowering women with this type of information is a very effective intervention [21].

Against this background, the aim of the present study was to investigate women’s perceptions of menstrual healthcare in rural Honduran communities. We focussed on the following objectives: (i) to ascertain the views of female rural community members in Honduras with regard to menstrual health education, (ii) to investigate the obstacles or difficulties that female community members may face due to their menstrual health, such as limited access to menstruation products or healthcare, and (iii) to investigate the attitudes within the community to menstruation.

## 2. Materials and Methods

In order to achieve our research aim, we adopted a statement-based survey study design. The study was conducted in the Potrerillos community, which is located in the municipality of Teupasenti, while a mobile medical clinic was offered to the community by the Global Brigades. Rapid Needs Assessment Reports are conducted by Global Brigades to identify new communities and to ascertain that Brigades will be possible to implement in the community. These data was accessed from the Monitoring & Evaluation Team at Global Brigades.

The Global Brigades’ ‘Rapid Needs Assessment’ report, published in February 2017, reported that Potrerillos has a population of 700 with 150 homes. In total, 15.3% of homes have latrines and 20% have either a bath or shower. The community has access to a Kindergarten and Middle School, where children are educated up to 9th Grade (14–15 years of age). The community has a local health centre (CESAR Potrerillos), where the most common diseases reported in adults are respiratory illness, fever and diabetes and arterial hypertension [22].

This study was approved by the School of Pharmacy, Safety and Ethics Subcommittee, University of Birmingham, UK, and by Global Brigades, Honduras; it was performed according to the Declaration of Helsinki. It was carried out over a period of 3 days between 16 and 18 August 2022. Participants were recruited as volunteers from amongst the female community members who attended the medical clinic in Potrerillos and showed interested in the research project. They were restricted to those over the age of 18 and under the age of 55, so as to collect data from women who had recently menstruated, and with the intention of excluding those who had gone through menopause. As the participants were taken from one rural community, simple random sampling was utilised as the sampling strategy. As participation was restricted to those attending the clinic, this presented a potential selection bias in the population. Participants received no payment for participation. As the study was a pilot study, no formal statistical power calculation was performed [23].

The study was completed with the assistance of two interpreters, who assisted recruitment and survey completion. The corresponding author provided the consent forms and surveys to participants and, with the assistance of the interpreters, provided an oral explanation of the study’s aims. An information sheet provided the participants with an explanation of the research aims and how the data collected would be used. The information sheet, consent form and survey were translated into Honduran Spanish with assistance from Global Brigades Honduras and the interpreters assisted by answering any further questions, or by explaining and reading aloud the information sheet. All participants signed or gave a fingerprint signature to confirm their consent and were informed of their right to withdraw at any time. All data collected were anonymised, as emphasised on the consent form. The presence of the interpreters and the information sheet aimed to reduce social desirability bias, by explaining the research aims to participants and by explaining the anonymisation of responses. Community volunteers, working with Global Brigades as members of the Potrerillos community, assisted with participant recruitment, directing female participants to the study and explaining the study objectives. This course of action aimed to increase participation and reduce selection bias by reaching as many potential participants as possible.

In total, 73 participants completed the 10-statement survey shown in (Table 1) which was designed to be completed using a Likert Scale (1–5), from strongly disagree to strongly agree. A participant number of 73, from an estimated population of 700, was considered sufficient to perform the pilot study, as it represented over 10% of the eligible population (women aged between 18 and 55 years of age) [24]. The statements were linked to the research objectives as follows: Statements 2, 3 and 4 assessed the extent of menstrual health education available to participants at and around the age of menarche. Since the aim was not only to explore menstrual health education, but also access to menstrual hygiene products and community attitudes to menstruation in rural Honduras, Statements 1, 7, 8 and 9 provided information on access to menstrual health products, while Statements 5, 6 and 10 investigated attitudes to menstruation within the community. The survey investigated the participants’ experience of menstrual education and attitudes throughout their menstrual history, from their first menstrual period, to their most recent. The survey was developed through reviewing the relevant literature, which quantitively investigated (i) sources of menstrual information and knowledge before menarche, (ii) menstrual product access and (iii) rates of ‘negative’ reactions to menarche or menstruation and social impacts [5], and by adopting the UK Government advice [25]. Due to the limited time the researchers of this study had in the rural community, it was not possible to validate the survey on a smaller, representative group of the study population. In anticipation of this, the survey statements were reviewed by Global Brigade Honduran workers and, prior to the initial data collection, discussed with the community volunteers and interpreters. The aims of these discussions were to ensure the survey contained short, simplistic statements that would allow a community with minimal to no exposure to surveys and data collections to adequately understand the statements, with the assistance of translators. A neutral option (3 = neutral) was included to potentially reduce response bias, allowing participants to ‘opt out’ of statements they were unsure of or uncomfortable answering.

Data Analysis: All data were uploaded into Excel spread sheets (Version 16.75), with individual participants anonymised and identified by numbers. For statements left blank or answered indecipherably, the spread sheet cell was left blank. Due to the pilot nature of this study, data analysis was limited to descriptive statistics and precision estimates, with no inferential statistics performed [23]. The data for each statement were summarised as percentages and the median and interquartile range (IQR), and, using PRISM (Version 10.4.1), 95% confidence intervals (CIs) for the median were calculated for each statement [26]. The percentages for each possible answer represented the ratio of participants selecting the answer out of the total participants answering the survey. The median reports the central tendency, or the most likely answer selected by participants [27]. The IQR represents the middle 50% of values and measures the spread of data [28]. Reliability of the survey results was assessed by Cronbach’s alpha using SPSS (Version 30.0.0.0).

## 3. Results

A total of 73 participants undertook the study, having given informed consent. Missing values and the response rate for each statement are shown in Table 2 and labelled ‘blank’ responses. Statements 6 and 9 were the statements with the highest number of blank answers, each with five missing participant responses. The average number of “non-blank” participant responses across the 10 statements was 70 (69.9).

**Figure 1:**
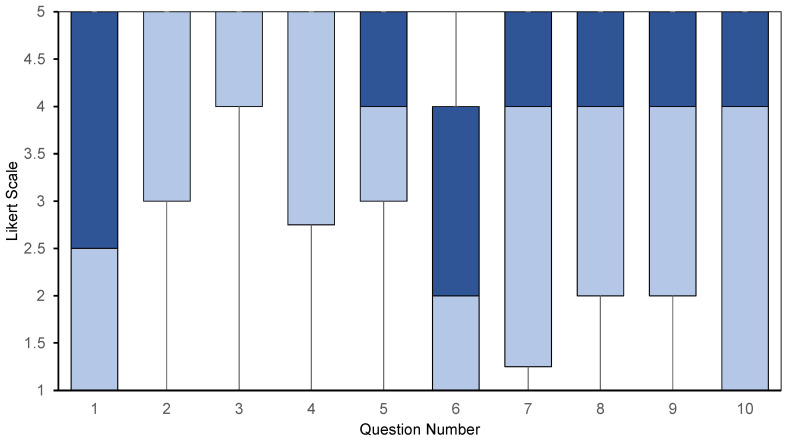
Box and whisker plot showing median and IQR of responses for each statement. For each statement, median, and 1st and 3rd quartiles are presented: dark blue represents the difference between the 3rd quartile and median, while light blue represents the difference between the median and 1st quartile. In each case, IQR is represented by the length of the box. Whiskers show values of maximum and minimum responses (1 and 5, respectively).

**Figure 2:**
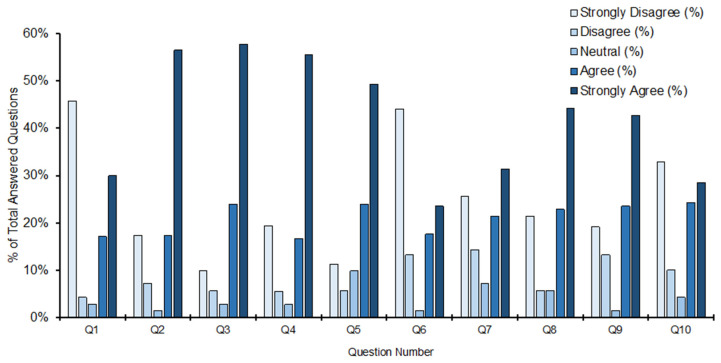
Percentage of each Likert scale response for each statement on the survey. Data for each of the 10 statements are shown on the horizontal axis. Columns for each statement represent the percentage of each score on the Likert scale, expressed as a percentage of the total responses for that statement (vertical axis). The score on the Likert scale shown by the intensity of shading (see key).

The descriptive statistics, median and IQR, are shown in Table 3. Box and whisker plots for the median and first and third quartiles are shown in Figure 1, while Figure 2 provides a bar chart indicating the percentages of each Likert scale answer for each statement.

### 3.1. Views of Female Rural Community Members in Honduras on Access to Menstrual Health Education (Statements 2, 3 and 4)

Statement 2, ‘Your understanding of menstrual health and management comes from explanations at home’, showed a median of 5 with an IQR (Q3–Q1) of 2 (97.05% CI: 4, 5). A majority (56.5%) of participants answered ‘strongly agree’, with 73.9% of participants selecting ‘agree’ or ‘strongly agree’. Only 24.4% of participants answered that they ‘strongly disagreed’ or ‘disagreed’, with 1.4% answering ‘neutral’ (Figure 2). For statement 3, ‘The menstruation and menstrual health management education you received in school means you are confident about your own menstrual health’, the median was 5, with an IQR of 1 (96.81% CI: 4, 5). The percentages of participants answering ‘strongly agree’ and ‘agree’ were 57.7% and 23.9%, respectively, only 15.5% of participants answering ‘strongly disagree’ or ‘disagree’. Finally, for statement 4, ‘You knew about menstruation and menstrual health before your first menstrual cycle’, the median was 5, with an IQR of 2.25 (95.56 CI: 4, 5). A majority (55.6%) of participants answered ‘strongly agree’, 16.7% answered ‘agree’, and 25% answered either ‘strongly disagree’ or ‘disagree’ (Figure 2).

### 3.2. Obstacles or Difficulties That Female Community Members Face Due to Limited Access to Menstruation Products or Healthcare (Statements 1, 7, 8 and 9)

Statement 1, ‘Your most recent menstrual cycle caused you to miss at least one day of work/school/normal demands of daily life’ reported a median of 2.4, with an IQR of 4 (95.86% CI: 1, 4) (Figure 1). The most frequent answer was ‘strongly disagree’ (45.7%), the second most frequent being ‘strongly agree’, with 30–50% of participants in total answering either ‘disagree’ or ‘strongly disagree’. However, a total of 47.1% of participants answered either ‘agree’ or ‘strongly agree’ (Figure 2). Further, for statement 7, ‘There is a lack of suitable and affordable menstrual health hygiene materials e.g., tampons, sanitary towels, within your community’, the median was 4, with an IQR 3.75 (95.86% CI: 2, 4), the most frequent answer being ‘strongly agree’ (31.4%), followed by ‘strongly disagree’ (25.7%). A total of 52.8% of participants answered either ‘agree’ or ‘strongly agree’ (Figure 1 and Figure 2).

Similarly, for statement 8, ‘There is a lack of choice in menstrual health hygiene materials e.g., tampons, sanitary towels, menstrual cups, within your community’, the participants reported a median of 4, with an of IQR 3 (95.86% CI: 4, 5); the most frequent answer was ‘strongly agree’ (44.3%), followed by ‘agree’ (22.9%). Only 21.3% of participants answered ‘strongly disagree’. Finally, for statement 9, ‘If you have pain or worries associated with your periods you have easy access to medication and/or healthcare’, the median was 4, with an IQR of 3 (96.15% CI: 4, 5), the most frequent answer being ‘strongly agree’, with a frequency of 42.6%. A total of 32.3% of participants answered either ‘strongly disagree’ (19.1%) or ‘disagree’ (13.2%) (Figure 2).

### 3.3. Attitudes Within the Community to Menstruation (Statements 5, 6 and 10)

For statement 5, ‘Attitudes towards menstruation and menstrual health management within your community are mostly positive’, the median was 4, with an IQR of 2 (96.81% CI: 4, 5), the most frequent answer being ‘strongly agree’ (49.3%), and a total of 73.2% answering either ‘strongly agree’ or ‘agree’. Only 11.3% and 5.6% of participants answered ‘strongly disagree’ and ‘disagree’, respectively, with 9.9% answering ‘neutral’. For statement 6, ‘You have been made to feel afraid and/or embarrassed about your menstrual cycle by other members of your community’, the median was 2, with an IQR of 3 (96.15% CI: 1, 4). The most frequent answer was ‘strongly disagree’ (44.1%), followed by ‘strongly agree’ (23.5%), making up a total of 17.6% and 13.2% answering ‘agree’ and ‘disagree’, respectively (Figure 2).

Finally, for statement 10, ‘You have experienced anxiety about your next menstrual cycle’, with reported a median of 4 and an IQR of 4 (95.86% CI: 2, 4), the most frequent answers were ‘strongly disagree’ and ‘strongly agree’, with frequencies of 32.9% and 28.6%, respectively. This resulted in a total of 52.9% of participants answering either ‘agree’ or ‘strongly agree’, although 42.9% answered ‘disagree’ or ‘strongly disagree’.

### 3.4. Reliability Analysis

Cronbach’s alpha was used for each objective to test the reliability of the survey results (Table 4). For around 10 items, a value of 0.6–0.7 is acceptable, indicating high internal consistency in responses [29]. Cronbach’s alpha for the whole survey was 0.605, demonstrating acceptable internal consistency. As each objective contained fewer than 10 items, a Cronbach’s alpha of >0.5 would indicate acceptable reliability [30]. For ‘views of female rural community members in Honduras on access to menstrual health education’, with three statements, the Cronbach’s alpha was 0.632, indicating acceptable reliability. For ‘obstacles or difficulties that female community members face due to limited access to menstruation products or healthcare’, the Cronbach’s alpha was 0.464. However, removing statement 8, ‘there is a lack of choice in menstrual health hygiene materials e.g., tampons, sanitary towels, menstrual cups, within your community’, raised the Cronbach’s alpha to 0.594, and therefore responses from statement 1, 7 and 9 for this objective showed acceptable reliability. For ‘attitudes within the community to menstruation’, the Cronbach’s alpha was 0.319, which indicates low reliability. Therefore, the results for the objective of ‘attitudes to menstrual health’ should be interpreted cautiously.

## 4. Discussion

This pilot study highlights important concerns for menstrual health management in the rural community of Potrerillos, indicating that amongst women who are resident in rural Honduras and between menarche and menopause, menstrual health education is weak and dependent on education in the home, with relatively poor access to menstrual healthcare products, restricted access to appropriate healthcare and concern about community attitudes to menstruation. In discussing these findings below, we have suggested how the deficits might be alleviated.

The most notable finding is that 25% of participants indicated they were somewhat unaware of menstruation and menstrual health before their first period. This is important because access to menstrual health education prior to menarche is crucial to prepare girls and to minimise misinformation or feelings of shame and embarrassment [20]. The present study indicated that the majority of participants received their menstrual health education at home (73.9%), and that some felt that the education given at school was inadequate to prepare them for menstruation. Thus, the introduction of menstrual health education in schools, focussing on menstrual health and management, could help to combat the reliance on at-home education. Certainly, an intervention study in Bangladesh reported significant improvements in menstrual health knowledge and practises, such as the use of sanitary pads, after the completion of 12 menstrual health educational seminars [31]. Additionally, teaching on puberty in a trial in Ghana improved school attendance of girls during their menstruation significantly [5]. Early exposure to menstrual health education before menarche can provide information to prevent discomfort and encourage good menstrual hygiene and school attendance. As previously reported, reliance on home education impacts the content and quality of menstrual health education. Standardised educational sessions provided by the community could minimise these disparities. A location such as Potrerillos, where community initiatives with Global Brigades are now widely accepted, would be a suitable location to introduce these sessions.

As far as menstrual healthcare and products are concerned, despite some variability amongst participants, the present study indicated that 52.8% of participants feel there is a lack of access to affordable menstrual products, with 67.2% indicating a lack of choice for different products. Further, 47.1% of participants admitted missing a day of school, missing work or not fulfilling the demands of daily life due to their most recent menstrual period. Thus, the provision of free, or cheaper menstrual health products is a potential solution to tackle this problem. However, it should be acknowledged that the implications on the local environment and waste management would need to be closely considered and that more sustainable options, such as menstrual cups, may be more appropriate. Indeed, a recent review on adolescent sexual and reproductive health stressed the importance of avoiding overreliance on the provision of menstruation products as a method of improving menstrual health in middle- and low-income countries, without a concomitant inclusion of holistic measures [32]. Thus, any increase or focus on improving access to products should be implemented together with educational initiatives and community measures so as to tackle stigma.

Turning to medical care as a way of targeting menstrual health, 32.3% of participants indicated restrictions to healthcare and medication access. The present study focussed on whether participants had ‘easy access’. However, it should be noted that studies performed in middle- and low-income countries have reported an ‘unwillingness’ to access healthcare even if it is available [5]. If this issue was relevant to the participants of the present study, it may have impact on interpretation of the results. For example, a study published in 2013, 218 girls in Brazil reported that 28.14% suffered from dysmenorrhea, with 51.9% of them rating their pain as ‘severe’, and yet only 13.39% sought medical assistance [33]. Similarly, a cross-sectional study in Ethiopia reported that only 9.8% of girls with severe dysmenorrhea sought medical care [34].

Considering attitudes to menstruation, the present study found that 16.9% of participants disagreed to some extent with the statement that attitudes to menstruation in their community were mostly positive, and a further 41.1% indicated they had been made to feel afraid or embarrassed about their menstrual cycle. This indicates that menstrual health stigma is present in the Potrerillos community. In addition, 52.9% responded that they had experienced anxiety about their next menstruation. Future studies should explore potential reasons or triggers for menstruation-associated anxiety, to investigate whether this is interlinked with inadequate product supplies or attitudes from others.

If the argument is accepted that menstrual health is a part of overall health, then women and girls should be free from any discrimination and stigmatisation surrounding menstrual health [2]. Thus, further investigation should be carried out in Potrerillos, and similar communities, into the circumstances of embarrassment of, fear of or negative associations with menstrual health so as to assess whether specific measures might be introduced to increase community education and dialogue surrounding menstrual health with the aim of improving the outcomes. It may be noted that misinformation can be a cause of negative associations with menstrual health, with 82% of girls in rural Nepal believing menstruation to be a curse [35]. Overall, community-led education sessions provided to community members, contingent with the provision of free menstrual hygiene products, have the potential to improve menstrual health hygiene in rural Honduras.

## 5. Limitations

This study attracted fewer participants than anticipated (the aim was to recruit 200 participants). This was due in part to the early closure of the medical clinic on day 1 caused by a medical emergency. Additionally, the COVID-19 pandemic prevented travel between communities, and therefore responses were collected from the Potrerillos community only. In the future, similar studies should be performed in other rural communities in Honduras to establish whether the present findings can be extrapolated.

Analysis of the data collected in the present study is limited by central tendency bias, social desirability bias and presented acquiescence bias, due to its reliance on a Likert scale to collect data [36]. Two translators facilitated explanations to participants who required it, and for those who were illiterate. However, we recognise that use of the Likert scale is an intrinsic limitation as it is unable to measure and test the extent to which participants understood each statement. For example, one participant included answered every statement as 5, ‘strongly agree’, despite the purposeful inclusion of reversal statements. These data were included in the analyses because there seemed to be no reason to exclude them given the reversal statements were exploring different issues: statement 5 assessed positivity on community attitudes towards menstrual health management, whereas statement 6 included a circumstance of personal fear or embarrassment. Further, although the values of Cronbach’s alpha indicated that the survey responses for the objectives relating to ‘views on access to menstrual health products’ and ‘obstacles faced due to limited access’ were generally reliable, those concerning ‘attitudes to menstrual health’ were unreliable. If the study were to be repeated, or used on a larger scale with greater resources, cognitive interviews in conjunction with a Likert scale survey would allow greater confidence in the results and ensure each participant understood every statement [37]. Interviews or focus groups would additionally allow for greater exploration into the answers. For example, the Likert scale indicated that anxiety surrounding upcoming menstrual periods is present in the community, but the survey was not designed to identify the most frequent or suspected cause of such anxiety. More detailed understanding of the reasons why participants hold their views could inform recommendations made on the basis of the findings. In addition, the short duration for which we were in the community limited the collection of information on personal characteristics of participants. Future studies should collect such data to allow further analyses based on age, ethnicity and socioeconomic factors. This would allow investigations of changes in education, product use and attitudes at the time of menarche based on the age of the participant.

## 6. Conclusions

The availability of menstrual health data in Honduras, particularly in rural locations, is limited. The broad aim of the present study was to assess women’s perceptions of menstrual healthcare in rural Honduran communities by performing a survey-based analysis, focussing on menstrual health education, access to menstrual hygiene products and community attitudes to menstruation. The findings from the present study suggest that the issues of inadequate menstrual health education, insufficient menstrual product access and limited access to medications and menstrual healthcare are present in the Potrerillos community of Honduras. In addition, the findings suggest negative associations with menstruation, through anxiety, or through being made to feel ashamed or embarrassed.

### Recommendations

These findings indicate a need for additional measures to address these problems through educational sessions, with a specific focus on menstruation for all community members, alongside an increase in the availability of supplies of menstrual health products. It seems likely that the present findings are representative of other similar rural communities in Honduras which raises the questions of whether a coordinated approach at government level, supported by the WHO or other organisations, could be particularly effective in improving menstrual and general health in Honduras. Future studies should focus on increasing participation numbers, potentially by visiting numerous rural communities, and should investigate the effects of age, ethnicity and socioeconomic status. Future non-profit healthcare initiatives, such as Global Brigades, should implement menstrual health education within their community outreach, facilitate provisions of menstrual hygiene supplies as part of their fundraising initiatives and take steps to monitor the effectiveness of the interventions. Future studies may wish, as indicated by this study, to conduct research on access to menstrual products by investigating which products (sanitary pads, menstrual cups or tampons) are preferred in the community, and what the most common replacements for a lack of products are (such as reusable cloths). This future research will be crucial to ensure that the provision of menstrual products to the community achieves the desired impact: women being provided with the hygiene products they are likely to use.

## Figures and Tables

**Table 1:** Statements used in the order in which they appeared on survey. Participants responded on a 5-point Likert scale from strongly disagree to agree (1–5, respectively).

	Statement
1	Your most recent menstrual cycle caused you to miss at least one day of work/school/normal demands of daily life.
2	Your understanding of menstrual health and management comes from explanations at home.
3	The menstruation and menstrual health management education you received in school means you are confident about your own menstrual health.
4	You knew about menstruation and menstrual health before your first menstrual cycle.
5	Attitudes towards menstruation and menstrual health management within your community are mostly positive.
6	You have been made to feel afraid and/or embarrassed about your menstrual cycle by other members of your community.
7	There is a lack of suitable and affordable menstrual health hygiene materials, e.g., tampons, sanitary towels, within your community.
8	There is a lack of choice in menstrual health hygiene materials, e.g., tampons, sanitary towels, menstrual cups, within your community.
9	If you have pain or worries associated with your periods you have easy access to medication and/or healthcare.
10	You have experienced anxiety about your next menstrual cycle.

**Table 2:** Number of blank (complete) and non-blank (missing) responses given by participants in statements. Total indicates the total number of respondents.

Statement	Non-Blank	Blank	Total	Response Rate (%)
1	70	3	73	95.9
2	69	4	73	94.5
3	71	2	73	97.3
4	72	1	73	98.6
5	71	2	73	97.3
6	68	5	73	93.2
7	70	3	73	95.9
8	70	3	73	95.9
9	68	5	73	93.2
10	70	3	73	95.9

**Table 3:** Median, interquartile range (IQR) and 95% confidence intervals (CI) of responses provided for each statement. The median measures the central tendency. The IQR measures the spread of data. CIs measure the degree of uncertainty of the estimates. The exact confidence levels are reported below in the results.

Statement	Median	IQR	95% CI
1	2.5	4	1, 4
2	5	2	4, 5
3	5	1	4, 5
4	5	2.25	4, 5
5	4	2	4, 5
6	2	3	1, 4
7	4	3.75	2, 4
8	4	3	4, 5
9	4	3	4, 5
10	4	4	2, 4

**Table 4:** Cronbach’s alpha for each objective, calculated using SPSS.

Test	Cronbach’s Alpha
10 Statements	0.605
Views of female rural community members in Honduras on access to menstrual health education (statements 2, 3 and 4).	0.632
Obstacles or difficulties that female community members face due to limited access to menstruation products or healthcare (statements 1, 7, 8 and 9).	0.464
Attitudes within the community to menstruation (statements 5, 6 and 10)	0.319

## Data Availability

The datasets used and/or analysed during the current study are available from the corresponding author on reasonable request.

## Abbreviations

The following abbreviations are used in this manuscript:

WHO	World Health Organisation
UN	United Nations
IQR	Interquartile Range
